# How ambient temperature affects mood: an ecological momentary assessment study in Switzerland

**DOI:** 10.1186/s12940-023-01003-9

**Published:** 2023-07-11

**Authors:** Marvin Bundo, Martin Preisig, Kathleen Merikangas, Jennifer Glaus, Julien Vaucher, Gérard Waeber, Pedro Marques-Vidal, Marie-Pierre F. Strippoli, Thomas Müller, Oscar Franco, Ana Maria Vicedo-Cabrera

**Affiliations:** 1 Institute of Social and Preventive Medicine, University of Bern, Bern, Switzerland; 2 Oeschger Center for Climate Change Research, University of Bern, Bern, Switzerland; 3 Graduate School for Health Sciences, University of Bern, Bern, Switzerland; 4 Department of Psychiatry, Psychiatric Epidemiology and Psychopathology Research Center, Lausanne University Hospital and University of Lausanne, Prilly, Switzerland; 5 National Institute of Mental Health, Bethesda, MD USA; 6 Department of Psychiatry, Division of Child and Adolescent Psychiatry, Lausanne University Hospital and University of Lausanne, Lausanne, Switzerland; 7 Department of Medicine, Internal Medicine, Lausanne University Hospital (CHUV), and University of Lausanne, Lausanne, Switzerland; 8 Department of Medicine and Specialties, Internal Medicine, Fribourg Hospital and University of Fribourg, Fribourg, Switzerland; 9 Translational Research Center (TRC), University Hospital of Psychiatry and Psychotherapy, University of Bern, Bern, Switzerland; 10 Privatclinic Meiringen, Bern, Switzerland; 11 Julius Center for Health Sciences and Primary Care, University Medical Center Utrecht, Utrecht, the Netherlands

**Keywords:** Climate change, Ambient temperature, Mental health, Mood, Ecological momentary assessment

## Abstract

**Background:**

Recent research has suggested that an increase in temperature can negatively affect mental health and increase hospitalization for mental illness. It is not clear, however, what factors or mechanisms mediate this association. We aimed to (1) investigate the associations between ambient temperatures and bad daily mood, and (2) identify variables affecting the strength of these associations (modifiers) including the time, the day of the week and the year of the mood rating, socio-demographic characteristics, sleep quality, psychiatric disorders and the personality trait neuroticism in the community.

**Methods:**

Data stemmed from the second follow-up evaluation of CoLaus|PsyCoLaus, a prospective cohort study conducted in the general population of Lausanne (Switzerland). The 906 participants rated their mood level four times a day during seven days using a cell phone app. Mixed-effects logistic regression was used to determine the association between daily maximum temperature and mood level. Participant ID was inserted as a random effect in the model, whereas the time of the day, the day of the week and the year were inserted as fixed effects. Models were controlled for several confounders (socio-demographic characteristics, sleep quality, weather parameters and air pollutants). Stratified analyses were conducted based on socio-demographic characteristics, sleep quality, presence of psychiatric disorders or a high neuroticism.

**Results:**

Overall, the probability of having a bad mood for the entire day decreased by 7.0% (OR: 0.93: 95% CI 0.88, 0.99) for each 5 °C increase in maximum temperature. A smaller and less precise effect (-3%; OR: 0.97: 95% CI 0.91, 1.03) was found when controlling for sunshine duration. A higher association was found in participants with bipolar disorder (-23%; OR: 0.77: 95% CI 0.51, 1.17) and in participants with a high neuroticism (-13%; OR: 0.87 95% CI 0.80, 0.95), whereas the association was reversed for participants with anxiety (20%; OR: 1.20: 95% CI 0.90, 1.59), depression (18%; OR: 1.18 95% CI 0.94, 1.48) and schizophrenia (193%; OR: 2.93 95% CI 1.17, 7.73).

**Conclusions:**

According to our findings, rising temperatures may positively affect mood in the general population. However, individuals with certain psychiatric disorders, such as anxiety, depression, and schizophrenia, may exhibit altered responses to heat, which may explain their increased morbidity when exposed to high temperatures. This suggests that tailored public health policies are required to protect this vulnerable population.

**Supplementary Information:**

The online version contains supplementary material available at 10.1186/s12940-023-01003-9.

## Background

There is increasing evidence that heat exposure has an impact on mental health. Recent studies have shown that increasing ambient temperatures are positively associated with increased psychiatric morbidity, suicides and mortality associated with psychiatric disorders [1]. However, little is known about the effects on mental health in the general population on a daily basis, on less severe symptoms rather than severe health outcomes such as hospitalizations or mortality, nor is it known whether demographic or personal characteristics of the individuals may influence this association [1].

Daily mood has been proposed as a potential mediator in the association between mental health and ambient temperature [2]. Daily mood evaluation can be used to predict the future mental health status of individuals and health outcomes [3]. It is a marker that can be measured on a daily basis and can help track minor changes in the mental status of the individuals. Previous studies suggested that ambient temperature can have an impact on stress levels, cooperation, aggressiveness and energy levels in individuals [4–6]. However, contradictory findings have been obtained on how mood is affected by temperature and whether this would depend on the mental conditions of the individual, with some studies suggesting that increased temperatures lead to worse mood [7, 8], while other studies show the contrary [1, 6, 9] or no effect at all [10]. However, these studies present several limitations: studies in experimental conditions which might not be representative of the general population-based impacts [11], the presence of recall bias in retrospective studies [8], single-day mood measurements, not considering the personal characteristics of study participants or not controlling for other environmental factors [7]. The use of more appropriate study designs (adequate methods for repeated measures in the same individual) or advanced statistical methods that allow the integration of the individual characteristics would help address these limitations and provide more robust evidence on the association between ambient temperature and mood states.

In the coming years, we will be faced with the challenges of unequivocal climate change. Now there is clear empirical evidence that human health is and will continue to be hugely impacted by increasing temperatures and other weather extremes [12]. Mental health patients seem to be particularly vulnerable to these conditions [1]. On the other hand, according to the World Health Organisation (WHO), mental health conditions are increasing worldwide (13%), accounting for one in five years lived with disability and projected to be one of the leading causes of disease burden by the end of this decade [13]. Therefore, it is critical to deepen our understanding of the effects of ambient temperature not only on more severe outcomes such as mental health morbidity or mortality, but also on more lenient outcomes such as daily mood variations, which can help predict and play a crucial role in the onset and maintenance of psychiatric conditions, both in the general population and among patients with existing mental health problems. This would provide public health policymakers with adequate tools to better predict, promote and protect population health from the effects of a changing climate.

Using a population-based sample we aimed to (1) investigate the associations between maximum ambient temperatures and daily bad mood, and (2) identify variables affecting the strength of these associations (modifiers) including the time, the day of the week and the year of the mood rating, socio-demographic characteristics, sleep quality, psychiatric disorders and the personality trait neuroticism.

## Methods

### Study sample

Data for the present paper stem from the second physical follow-up of CoLaus|PsyCoLaus [14, 15], a prospective population-based study based on an original sample of 6,734 individuals aged 35 to 75-year-old randomly selected between 2003 and 2006 from the population of the city of Lausanne (Switzerland) according to the civil register. The study was designed to investigate cardiovascular risk factors and mental disorders as well as their associations in the community. At the second follow-up information on daily mood was collected through an ecological momentary assessment (EMA) tool as part of the physical evaluation (2015–2017).

**Fig. 1:**
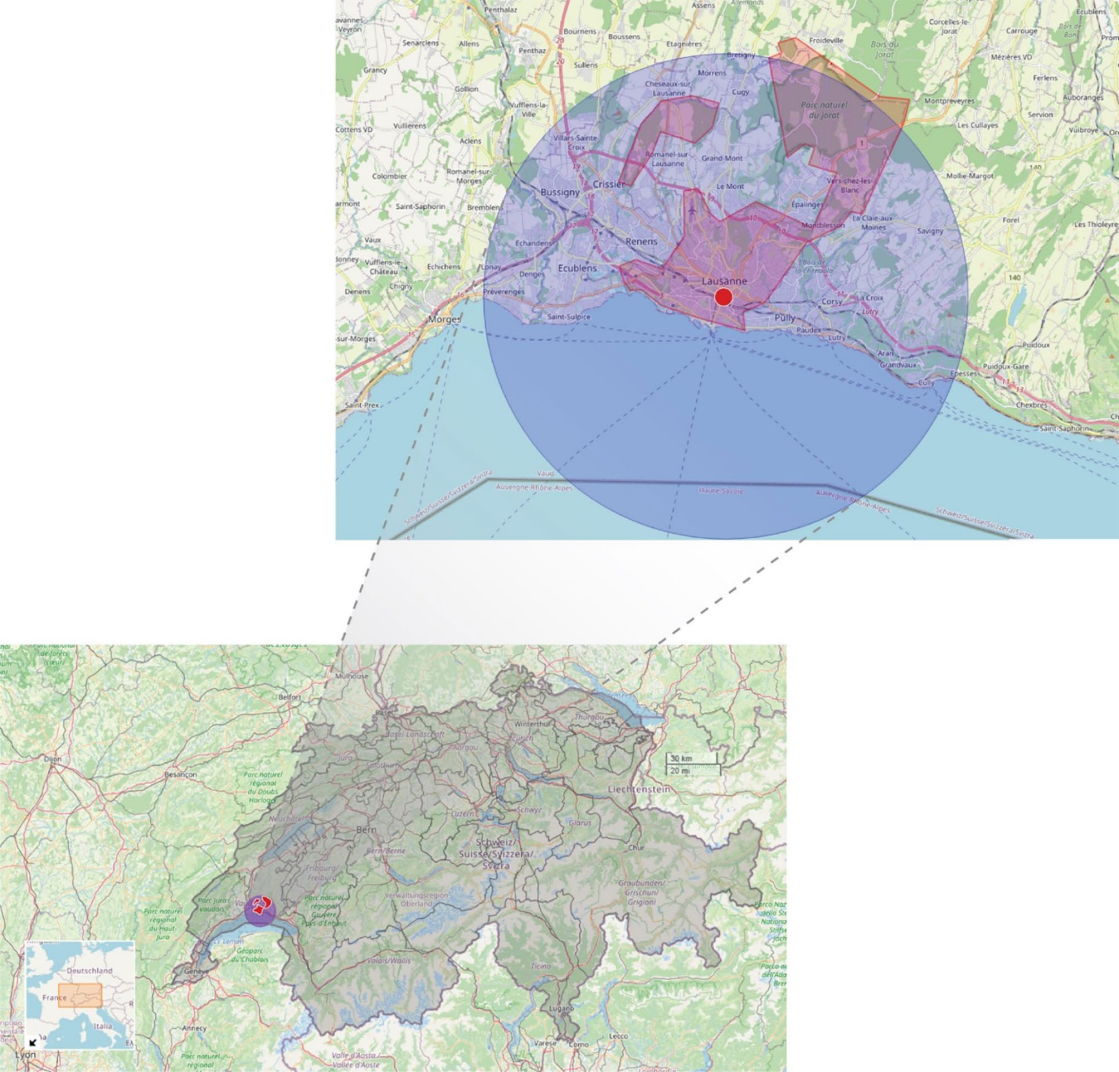
Location of the weather station and the 10-km radius buffer zone around the station

A valid measurement for mood was collected for 934 participants. To reduce bias due to exposure misclassification, for the present analyses we only included participants living within a 10-km radius of the NABLAU weather station in the city of Lausanne, from which we extracted the information on hourly average ambient temperature (Fig. 1), resulting in a sample of 906 participants. Among them, 789 participants took part in the psychiatric evaluation.

### Ecological momentary assessment (EMA)

EMA is a tool allowing researchers to repeatedly measure a person’s current behaviour and experience in real-time and in his/her natural environment [16]. It has been reported that this measurement tool minimizes recall bias, maximizes ecological validity, and allows for the study of micro-processes that influence behaviour in real-world settings [16]. In the present study, we relied on self-reported real-time assessments of the participants’ mood using cellular phones provided by the study team. Mood level was assessed during seven consecutive days at four different times of the day (morning 9:00 am, noon 12:00 pm, afternoon 4:30 pm and evening 9:00 pm). Each time, participants were asked to rate their mood level by answering the question “How do you feel today?”. The response was coded using a Likert scale ranging from one to seven. One represented the highest (extremely happy), and seven the lowest mood level (extremely sad) [17]. Rated mood levels were dichotomized by the median level (i.e., 2). Mood ratings of 3 and above were considered as bad mood. Data on sleep quality [Likert scale of one to seven (extremely good sleep quality to extremely bad sleep quality), only once in the morning] was also collected. It was assessed that 83% of all self-reported assessments per time of the day were successfully completed and therefore included in the analysis. The remaining 17% were unsuccessful due to a variety of factors such as missed assessments from the participants, canceled assessments (assessment was started but then abandoned by the participant) or unprocessed data due to device issues (i.e., power shortage). Only 3% (n = 27) of the participants reported uninformative daily mood measurements (same Likert scale score over the entire week).

### Environmental data

Daily data on hourly average ambient temperature (celsius) and other weather variables (relative humidity (%), sunshine duration in hours, rainfall (total sum of mm in one hour) and barometric pressure (hPa)) were obtained from the urban weather station NABLAU located in the city of Laussane (MeteoSwiss [18]. We collected average hourly measurements for all weather variables, except for the sunshine duration for which hourly measurements were not available. Daily average levels of particulate matter with a diameter less than 10 μm (PM_10_, µg/m^3^), nitrogen dioxide (NO_2_, µg/m^3^), and ozone (O_3_, µg/m^3^) were derived from hourly measurements registered in the Nabel station in the city of Laussane run by the Department of Industrial, Urban and Rural Environment in Vaud (DGE-DIREV).

### Covariates

We also collected information on age, sex, and socioeconomic determinants, including marital status (single or married), and level of education (two categories: low and medium + high; medium + high was defined as individuals attending high school, University or a similar institution) at the time of the recruitment.

### Psychological data

Diagnostic criteria for lifetime psychiatric disorders (schizophrenia and bipolar disorder) and current disorders (depression and anxiety) at the time of the EMA investigation were elicited using the French version of the semi-structured Diagnostic Interview for Genetic Studies (DIGS) [14, 18, 19]. This diagnostic interview was completed at each follow-up evaluation. Detailed information on mental disorders assessment through DIGS is presented in the supplementary file [see Supplementary File 1]. The personality trait neuroticism was assessed by the Eysenck Personality Questionnaire (EPQ) [20]. Participants categorized as currently experiencing depression were those who had been diagnosed with major depressive disorder, whereas participants identified as currently experiencing anxiety were those diagnosed with any one of the following anxiety disorders: agoraphobia, social phobia, panic disorder, or generalized anxiety disorder. A high level of neuroticism was defined as a score equal to or exceeding the 50th percentile.

### Statistical analysis

Multilevel random-effects linear regression models were used to investigate the association between maximum temperature and mood level. Using these types of models, the participant serves as his or her own control and they can adjust variations in repeated measurements for the same subject for the EMA data. The temperature was inserted as a fixed linear function in the model. The main exposure was defined as the maximum temperature (highest recorded hourly average temperature) from the four hours preceding each instance when a mood marker was recorded. In the main model, we also included random intercepts per individual to account for within-subject correlation that arises due to repeated measurements on the same subjects. Additionally, the following time variables were included in the model as a fixed effect based on the Akaike Information Criterion (AIC, a statistical measure used to compare the goodness of fit of different models for a given set of data): time of the day (morning, noon, afternoon and evening), day of the week and year when the mood measurement was conducted. The goodness of fit results are shown in Table S1 [see Supplementary File 1]. Different variables were considered as possible confounders in the model: socio-economic characteristics (age, sex, education, marital status), air pollutants (levels of particulate matter with a diameter less than 10 μm (PM_10_), nitrogen dioxide (NO_2_), and ozone (O_3_)) and other weather parameters (humidity, sunshine duration, rainfall and barometric pressure). For all the weather variables, except sunshine for each only daily measurements were available, an average measurement for the four hours preceding each mood assessment was calculated and used in the model. Based on the change in estimate of the main effect, the correlation between the parameter and temperature, and the AIC, only sunshine duration was selected as a potential confounder (Table S1) [see Supplementary file 1]). Therefore, we ran another set of models that included this variable with a linear function.

Several stratified analyses were conducted according to the characteristics of the participants (sex, age, marital status, level of education), sleep quality, the presence of current (depression, anxiety disorders) or lifetime (bipolar disorder, schizophrenia) psychiatric disorders and a high neuroticism, and for participants not diagnosed with any mental health disorder. Separate analyses were performed for weekdays and weekends, and by season (meteorological seasons: autumn, winter, spring, summer) of the year in which the data were collected. Tests for interactions were used to evaluate for statistically significant subgroup differences and temporal analyses.

To test the robustness of the model several sensitivity analyses were conducted. The association between temperature and mood level was assessed when using the mean and the minimum temperature instead of the maximum temperature. We tested for a potential non-linearity of the association by using a model where temperature was introduced as a non-linear function in the model (as a natural spline with knots at the 50th percentile and knots at the 50th and 90th percentile). Furthermore, a sensitivity analysis was performed by considering an alternative cutoff value of two (instead of three) for the dichotomized mood level. Finally, we conducted two additional sensitivity analyses including participants who lived within a narrower (4 km) or wider (20 km) radius from the weather station.

All the statistical analyses were conducted in R (version 3.4.4, R Development Core Team) using the packages lme4 and dlnm.

## Results

### Description of the sample

The description of the sample in terms of demographic factors, psychiatric disorders and neuroticism is presented in Table 1. In brief, 496 (54.7%) were women and 287 (31.7%) were older than 65 years. A total of 75.6% of the individuals had a medium or high level of education and 57.5% declared not to be married at the time of the survey. Major depressive disorder and any anxiety disorder at the time of the EMA measurement were reported by 10.0% and 5.1% of the participants respectively, while lifetime bipolar disorder and schizophrenia were rare (less than 3%).

The descriptive statistics for the weather variables are shown in Table 2. During the exposure time of the study participants (from 2015 to 2017), the median temperature for the radius area was 11.1 °C (25th-75th percentile: 6.1–17.8), and 4.5 h (0.4–5.3)  for sunshine duration.

**Table 1:** Description of the study sample

		N	%
Total		906	
Sex	Male	410	45.3
	Female	496	54.7
Age	< 65 years old	619	68.3
	≥ 65 years old	287	31.7
Marital status	Single (living without a partner)	510	57.5
	Married	377	42.5
Level of education	Low	221	24.4
	Medium or high	683	75.6
Mental disorders	Total*	789*	
	Major Depressive Disorder (current)	79	10.0
	Any anxiety disorder^a^ (current)	40	5.1
	Schizophrenia (lifetime)	7	1.0
	Bipolar disorder (lifetime)	19	2.4
Personality traits	Total	789*	
	A score above the 50th percentile (median) for neuroticism	395	50

* Data on psychiatric disorders and neuroticism were not available for 117 participants (12.9%). Data were also not available for 2 participants regarding education and for 19 participants regarding marital status

^a^ Anxiety disorder: agoraphobia, social phobia, panic disorder, general anxiety disorder

**Table 2:** Descriptive statistics for daily ambient temperature, other weather parameters, and air pollutants

Environmental factor	Minimum	25^th^ Pct	Median	Mean	75th Pct	Maximum
Temperature	-6.3	6.1	11.1	12.1	17.8	34.6
Sunshine duration (hours)	0.0	0.5	4.5	5.3	9.2	13.9
Precipitation (sum of mm per hour)	0.0	0.0	0.0	0.1	0.0	5.9
Relative humidity (%)	15.0	56.3	68.8	67.3	80.3	99.5
Pressure (hPa)	927.0	953.0	957.5	957.1	961.5	977.6
O3 (µg/m^3^)	1.4	22.9	43.1	41.4	57.8	104.6
PM10 (µg/m^3^)	2.4	9.1	13.7	14.8	18.7	81.0
NO2 (µg/m^3^)	11.2	33.0	42.1	41.9	50.3	76.4

*Pct = Percentile

### Association between ambient temperature and mood level

**Fig. 2:**
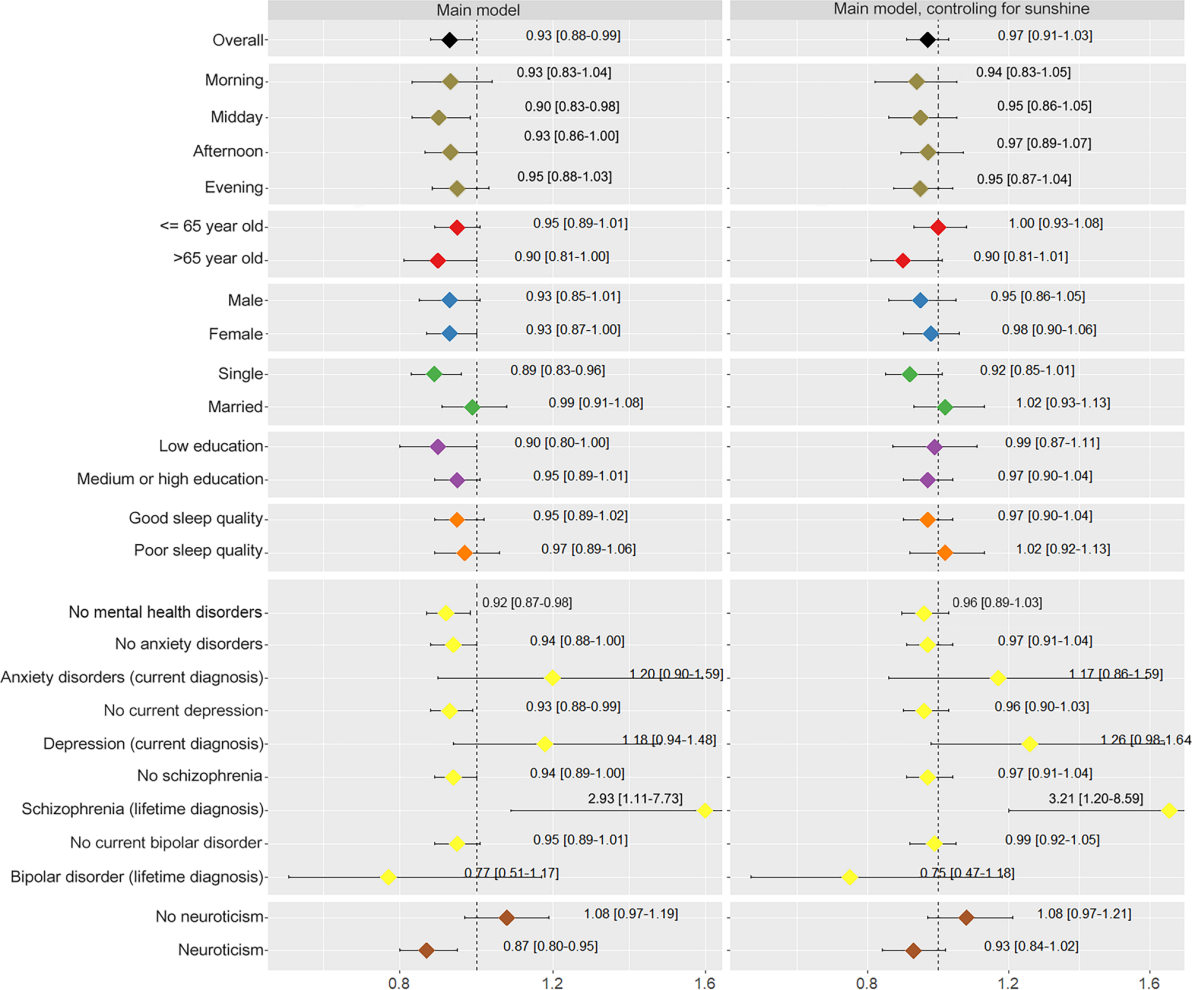
Overall and by subgroup association estimates of maximum temperature and bad mood

As shown in Fig. 2, overall, the probability of having a bad mood for the entire day decreased by 7.0% (OR: 0.93: 95% CI 0.88, 0.99) for each 5 °C increase in maximum temperature. When controlling for the sunshine duration, the association persisted, albeit with a smaller and less precise effect (-3%; OR: 0.97: 95% CI 0.91, 1.03).

In subgroup analysis (Fig. 2), a decrease of -10% was found in the elderly (OR: 0.90: 95% CI 0.81, 1.00) and of -5% in younger participants (OR: 0.95: 95% CI 0.89, 1.01); a decrease of -11% in single people (OR: 0.89: 95% CI 0.83, 0.96) and of -1% in married ones (OR: 0.99: 95% CI 0.91, 1.08); and a decrease of -10% in those with low education (OR: 0.90: 95% CI 0.80, 1.00) and of -5% in those with medium or high education (OR: 0.95: 95% CI 0.89, 1.01).

The probability of having a bad mood for the entire day decreased in individuals with a lifetime bipolar disorder (-23%; OR: 0.77: 95% CI 0.51, 1.17). A positive association was found in participants with a current diagnosis of anxiety (20%; OR: 1.20: 95% CI 0.90, 1.59), with a current diagnosis of depression (18%; OR: 1.18 95% CI 0.94, 1.48) and lifetime diagnosis of schizophrenia (193%; OR: 2.93 95% CI 1.11, 7.73). For participants scoring above the 50th percentile for neuroticism, a higher negative association was found between temperature and bad mood (-13%; OR: 0.87 95% CI 0.80, 0.95), whereas the result was reversed for participants scoring below the 50th percentile (8%; OR: 1.08 95% CI 0.97, 1.19). Among participants without any mental disorders, the estimate was similar to the main estimate for the entire population (8%; OR: 1.08 95% CI 0.87, 0.98).

Regarding the entire subgroup analyses, the interaction tests showed significant results for anxiety and neuroticism (p = 0.04 and p = 0.004 respectively, Table S2 [see Supplementary File 1].

The association between bad mood and maximum temperature was similar when analyzing each time of the day separately (Fig. 2), day of the week and season (Table S3) [see Supplementary File 1], although the results were less precise. The models assuming a non-linear relationship did not provide a better fit and the resulting curve was close to linear (Figure S1) [see Supplementary file 1]. When applying an alternative cuttoff value for the dichotomized mood level, the results remained robust for the main result and for the stratified analyses based on socio-demographic characteristics of the individuals. The results were less robust for the stratified analyses based on the presence of mental health disorders (Table S4) [see Supplementary File 1]. Additionally, the association remained similar when considering narrower or larger radiuses (Table S5) [see Supplementary File 1], and when using average or minimum temperature instead of maximum temperature (Figure S2) [see Supplementary File 1].

The model on the left was not controlled for sunshine duration, whereas the model on the right was additionally controlled for sunshine duration.

In the models, participant ID was inserted as a random effect, whereas the time of the day, day of the week and season of the year as fixed effects. A result below 1 means that an increase in 5ºC of maximum temperature reduces the probability of having a bad mood.

Anxiety disorder: agoraphobia, social phobia, panic disorder, general anxiety disorder.

High neuroticism: score equal to or above the 50th percentile.

## Discussion

Our findings indicate that overall increasing temperatures are associated with better mood in the general population. In particular, each 5 °C increase in maximum ambient temperature was associated with a 7% higher probability of having a good mood in the general population in Lausanne. The association was less precise and lower (3%) when controlling for the sunshine duration, suggesting that part of the effect of temperature might be mediated by the amount of sun during that day.

This pattern is consistent with previous studies conducted in other settings (in the United States and United Kingdom) which have assessed the association between ambient temperature and mood markers [6, 9, 21]. The association was found to be linear, consistent with studies that assessed the association between temperature and mental health outcomes (morbidity, mortality, and suicide) [1]. The possibility that warmer temperatures allow individuals to engage more in interpersonal and social interactions and leisure activities, as well as visit green and blue spaces, could explain this association. The absence of these activities can lead to more depressive feelings, while participating in them can improve mood [22–24].

Based on the subgroup analysis, although not reaching statistical significance, the result may indicate a higher association in older, single or less educated participants. There are mixed findings regarding the influence of demographic factors on mental health outcomes. For example, some studies suggest that male or old individuals are more susceptible to mental health morbidity when exposed to heat [25–27], whereas other studies suggest the contrary [28, 29], or no difference at all [30]. Noelke et al. suggested that age and education are important moderators of the temperature-mood association, whereas sex does not play any role [8]. This heterogeneity of results regarding the demographic factors as moderators could be explained by the difference in socioeconomic status, cultural background, and family history for each of these subpopulations. Within and between populations, these factors could indirectly influence susceptibility. Previous studies have shown that sleep quality is positively associated with a positive mood [31, 32]. However, our study showed that the quality of sleep does not influence the impact of ambient temperature on daily mood.

Our findings show that people with an anxiety disorder could be negatively influenced by increasing temperatures. A previous study in South Korea has shown that the risk for an emergency visit due to a panic attack increase by 2.2% for each one-degree increase in temperature [33]. Another study conducted in Barcelona found a positive association between hot wind and panic anxiety [33]. The relationship between increased ambient temperature and anxiety symptoms may be explained by serotonin system dysregulation, the effect of psychotropic medications, and physiological changes such as dehydration [33]. Increasing temperature temporarily lowers serotonin levels in the brain, which is associated with higher levels of anxiety and fear [33–35]. Drugs used to treat symptoms of anxiety, such as antidepressants or sedatives, can disrupt the thermoregulatory mechanisms in the body [33, 36]. Dehydration, caused by sudden changes in temperature, can reduce serotonin levels and affect the pharmacokinetics of psychotropic drugs [33, 37, 38].

Similarly, the mood of individuals who scored below the 50th percentile score in the EPQ test for neuroticism might be negatively influenced by ambient temperature. These findings contradict those of a study that showed that individuals with a neurotic personality respond to heat with “diminished sweat rate, reduced tolerance and greater discomfort” compared to the general population [39]. However, it should be noted that the study was not population-based and not based on the median score for neuroticism.

The large imprecision of the estimates for the other sub-diagnoses may be due to the small sample sizes and we believe that these results deserve discussion as well.

Our findings suggest that people with schizophrenia might react differently to increasing temperatures, being the subgroup in the analysis in which mood was mostly negatively influenced by the exposure. This is compatible with several studies indicating that these patients are sensitive to high temperatures which can lead them to increased morbidity, exacerbations, hospitalizations, and death rates [1, 40–43]. Also, a recent 45-year time-series study conducted in Bern, Switzerland, showed that schizophrenic patients are one of the most impacted groups by high temperatures (10.0% daily increase in daily hospitalizations rate for 10-degree ambient temperature increase; OR: 1.10: 95% CI 1.04, 1.15) [30]. The ability to cope with heat stress may be inadequate in these patients due to an impaired peripheral (“impaired heat loss through peripheral vasodilatation via abnormalities in niacin and PGE1”) and central thermoregulation system (“disruption of the mesolimbic dopamine system”) [44]. These patients are also prescribed neuroleptics which may affect their body’s ability to redistribute heat from its core to its periphery [44]. Other accompanying disease characteristics (for example high dependency on caregivers, poor general health, low economic status, etc.) could inhibit these individuals from taking advantage of engaging in social/physical activities like the general population when the temperatures increase [40, 45–47].

The indication for a higher-than-normal mood improvement in participants with bipolar disorder compared to the general population could possibly be explained by a switch of the manic state by high temperatures [48]. Accordingly, the reversed results of the association in participants with a current diagnosis of depression may relate to decreased platelet serotonin and increased levels of serotonin in the blood, which can be induced by exposure to heat [35].

The presence of mood disturbances is not only an indicator of mental health disorders but can also be a predictor of these illnesses and play an important role in influencing other factors that drive or maintain these conditions. Several studies have suggested that negative mood patterns may be able to predict the onset of depressive symptoms [49, 50], Additionally, mood can have an impact on a variety of psychological processes and behaviors that have an impact on one’s mental health. A negative mood state can lead to negative thinking patterns, self-criticism and ruminations, factors all of which can contribute to the onset and maintenance of mental health disorders [51, 52]. Cognitive or decision-making abilities can also be impaired by the presence of a negative mood. For example, individuals with depressed mood tend to be more focused on negative information or recall more often negative memories and get more easily overwhelmed when confronted with problems [53–55]. On the other hand, a positive mood has a protective role by enhancing resilience, promoting problem-solving skills, and improving cognitive abilities [56–58].

Climate change’s effects on mental health are multi-faceted, involving both indirect (through conflict and migration) and direct pathways (exposure to heat). Recent studies have shown that the impact of increasing temperatures is already evident even in countries with temperate temperatures like Switzerland for outcomes such as hospitalizations or suicide [30, 59]. However, this study shows that heat could possibly have an impact also on softer mental health outcomes such as daily mood. Mood disturbances can significantly degrade the quality of life and contribute to the onset and maintenance of more severe mental health conditions over time. According to climate change projections, the mental health toll of increasing temperatures is expected to increase in the future. Therefore, specific public health measures (education and awareness campaigns on the issue, early warning systems and emergency preparedness for heat, creation and preservation of green and blue spaces in urban areas, psychosocial support services for vulnerable groups, etc.) should be implemented to counteract this impact.

To our knowledge, this is the first study to assess the impact of ambient temperature on daily mood using participants from a large cohort population study. This is also the first time EMA has been used to answer this research question, a methodology well suited for repeated measures such as mood markers. A wide range of data was available for demographic factors, and individual or behavioural characteristics of the individuals, which allowed us to control for the role of these factors and to conduct several stratified analyses. The sensitivity analyses showed that the results were robust applying different modelling choices (non-linearity of the association, different weather station radiuses, and using maximum and minimum temperature instead of average temperature). Furthermore, this is the first study assessing the associations in subpopulations with specific psychiatric diagnoses.

However, this study presents several limitations. First, we assumed the same level of exposure to the environmental factors, including ambient temperature, for the entire study population resulting in potential biases due to exposure misclassification. That is, high-resolution environmental data was not available, which would have allowed us to assign an individual level of exposure to each participant. To reduce the impact of the exposure misclassification, the study included only individuals who lived within a limited radius of 10 km from the weather station. However, it should be noted that this would only affect the precision of the estimates. Additionally, sensitivity analyses showed that the results were robust when considering wider or narrower radiuses. Second, data on the presence of psychiatric sub-diagnoses were available only for certain diseases (anxiety, depression, schizophrenia and bipolar disorders), therefore the effect of temperature on mood could not be assessed for the entire spectrum of psychiatric conditions. Third, we did not have information on movement or activity patterns potentially relevant such as visiting blue or green spaces. Fourth, it was not possible to do a sub-group analysis based on the psychiatric drugs that the participants take. Fifth, there could be a “novelty seeking” selection bias regarding the participants who completed the EMA questionnaire. Finally, although individuals were recruited from a large cohort study, the study area included only the city of Lausanne. Considering the heterogeneity of associations between temperatures and health outcomes within countries and regions [60], the results may not be representative of the Swiss or European populations.

## Conclusion

According to our findings, rising temperatures may positively affect mood in the general population. In individuals with some psychiatric disorders or specific personality traits, however, this association seems to be reversed. People with psychiatric disorders may experience different responses to heat, which may explain their increased morbidity when exposed to high temperatures. With a changing climate, the negative impact of temperature on mental health of people with psychiatric disorders could be amplified in the future. This suggests that tailored public health policies are required to protect this vulnerable population (more resilient mental health institutions to heat, specific heat alerts and information for those patients, training of healthcare professionals on the topic etc.). This study paves the way for larger studies at a national or international level on the general population and psychiatric patients, for a better understanding of the physiological and pathophysiological mechanisms of how temperature influences mood and mental health morbidity.

## Electronic supplementary material

Below is the link to the electronic supplementary material.

Supplementary Material 1

## Data Availability

The data of CoLaus|PsyCoLaus study used in this article cannot be fully shared as they contain potentially sensitive personal information on participants. According to the Ethics Committee for Research of the Canton of Vaud, sharing these data would be a violation of the Swiss legislation with respect to privacy protection. However, coded individual-level data that do not allow researchers to identify participants are available upon request to researchers who meet the criteria for data sharing of the CoLaus|PsyCoLaus Datacenter (CHUV, Lausanne, Switzerland). Any researcher affiliated to a public or private research institution who complies with the CoLaus|PsyCoLaus standards can submit a research application to research.colaus@chuv.ch or research.psycolaus@chuv.ch. Proposals requiring baseline data only, will be evaluated by the baseline (local) Scientific Committee (SC) of the CoLaus and PsyCoLaus studies. Proposals requiring follow-up data will be evaluated by the follow-up (multicentric) SC of the CoLaus|PsyCoLaus cohort study. Detailed instructions for gaining access to the CoLaus|PsyCoLaus data used in this study are available at www.colaus-psycolaus.ch/professionals/how-to-collaborate/.
