# Supplementary material for: How ambient temperature affects mood: an ecological momentary assessment study in Switzerland
Source: Environ Health. 2023 Jul 11;22:52. doi: 10.1186/s12940-023-01003-9 (PMC10334623; doi:10.1186/s12940-023-01003-9)
Supplement: Supplementary file 1 — Supplementary Material 1 [file 12940_2023_1003_MOESM1_ESM.docx]

# **Supplementary files**

## Mental disorder assessment through Diagnostic Interview for Genetic Studies (DIGS)

Assessment of mental disorders (schizophrenia, bipolar disorder, depression and anxiety) was conducted using the semi-structured Diagnostic Interview for Genetic Studies (DIGS) [1-3]. Interviewers, who were all master-level psychologists or psychiatrists, underwent a comprehensive two-month training including a rating practice and co-supervised ratings, and were under the supervision of a senior psychologist.
The DIGS was created by the National Institute of Mental Health Molecular Genetics Initiative (NIMH) to facilitate a more accurate evaluation of phenotypes encompassing a broad range of DSM-IV Axis I criteria including mood, psychotic disorders, anxiety disorders and substance use.

The original English [3] and French versions [2] of this instrument underwent thorough reliability testing for various disorders, including psychosis, mood disorders, and substance use disorders. In the French version, inter-rater and test-retest reliability for major depressive disorder (MDD) showed kappa values of 0.93 and 0.62, respectively[4].

Furthermore, as the original DIGS lacked questions for the assessment for generalized anxiety disorder (GAD), it was completed with sections for GAD and phobias from the French version of the Schedule for Affective Disorders and Schizophrenia - Lifetime and Anxiety Disorder Version (SADS-LA) [5]. The French translation of the SADS-LA demonstrated satisfying test-retest reliability for panic disorder/agoraphobia (Yule's Y = 0.43), GAD (Yule's Y = 0.61), and phobic disorders (Yule's Y = 0.66).

The Statistical Manual of Mental Disorders (DSM IV) criteria were used to determine the diagnostic classification [6]. The following grouping was used for the Main Axis disorders:

1. Major Depressive disorders (MDD) and bipolar disorder (bipolar I and II).

2. Anxiety disorders (agoraphobia, social phobia, panic disorder, or generalized anxiety disorder).

3. Psychotic disorders (schizophrenia, brief psychotic disorders, schizophreniform disorder, and schizoaffective disorder).

4. Alcohol use disorders.

5. Drug use disorders.

**Table S1. Selection of model parameters.**

| **Selection of the time variables (AIC results)** | | |
| --- | --- | --- |
| **Parameters used in the model** | **Model estimate** | **AIC** |
| ID, temperature | 0.76 [0.73-0.81] | 21323.65 |
| ID, temperature, time of the day | 0.93 [0.88-0.98] | 20880.42 |
| ID, temperature, time of the day, study day | 0.93 [0.88-0.98] | 20802.74 |
| ID, temperature, time of the day, study day, year* | 0.93 [0.88-0.99] | 20800 |
| ID, temperature, time of the day, study day, year, season | 0.95 [0.90-0.1.01] | 20802.66 |
| **Selection of the time-varying variables (correlation)** | | |
| **Parameter** | **Correlation maximal temperature - parameter** | |
| Sunshine duration** | 0.61 | |
| Relative humidity** | -0.62 | |
| Barometric pressure | -0.05 | |
| Precipitation | -0.03 | |
| NO2 | -0.08 | |
| PM10 | -0.07 | |
| O3 | 0.06 | |
| **Selection of the time-varying variables (AIC results)** | | |
| ID, temperature, time of the day, study day, year, season + humidity | 0.96 [0.91-0.1.03] | 20797.17 |
| ID, temperature, time of the day, study day, year, season + sunshine duration*** | 0.97 [0.91-0.1.03] | 20795.37 |
| ID, temperature, time of the day, study day, year, season + humidity + sunshine duration | 0.98 [0.92-0.1.05] | 20796.17 |

*Based on the AIC results, the following time variables were selected to be included in the model: temperature, time of the day, study day and year.

**Based on the correlation tests results, from all the time-varying variables, only humidity and sunshine duration were initially considered to be included in the model.

***Based on the AIC results, only sunshine duration was considered a potential confounder and included in the model.

**Table S2. Results of the interaction tests for the different parameters**.

| **Parameter** | **Interaction test p value for main model** | **Interaction test p value for main model + controlling for sunshine** |
| --- | --- | --- |
| **Sex** | 0.45 | 0.43 |
| **Age** | 0.40 | 0.37 |
| **Education** | 0.48 | 0.47 |
| **Marital status** | 0.08 | 0.09 |
| **Sleep quality** | 0.25 | 0.25 |
| **Anxiety** | 0.04 | 0.04 |
| **Depression** | 0.08 | 0.08 |
| **Bipolar disorder** | 0.34 | 0.34 |
| **Schizophrenia** | 0.08 | 0.09 |
| **Neuroticism** | 0.004 | 0.004 |

**Table S3. Temporal analyses by day of the week and season.**

|  | **Temporal analysis by:** | **Odd Ratio [95% Confidence interval]** |
| --- | --- | --- |
| **Day of the week** | Weekend | 0.96 [0.87-1.06] |
|  | Weekday | 0.93 [0.88-1.00] |
| **Season** | Winter | 0.92 [0.78-1.09] |
|  | Spring | 0.93 [0.81-1.06] |
|  | Summer | 0.92 [0.83-1.03] |
|  | Autumn | 0.95 [0.83-1.08] |

**Table S4. Sensitivity analysis by considering an alternative cutoff value (two) for the dichotimized mood level.**

| **Parameter** | **Odd Ratio [95% Confidence interval]** |
| --- | --- |
| **Overall** | 0.92 [0.86-0.99] |
| **Morning** | 1.03 [0.86-1.24] |
| **Midday** | 0.83 [0.74-0.93] |
| **Afternoon** | 0.86 [0.77-0.95] |
| **Evening** | 0.95 [0.86-1.05] |
| **Young** | 0.95 [0.88-1.04] |
| **Old** | 0.85 [0.75-0.96] |
| **Male** | 0.93 [0.84-1.04] |
| **Female** | 0.91 [0.83-1.00] |
| **Single** | 0.91 [0.83-0.99] |
| **Married** | 0.94 [0.84-1.06] |
| **Low education** | 0.81 [0.71-0.92] |
| **Medium or high education** | 0.96 [0.89-1.05] |
| **Good sleep quality** | 0.92 [0.85-1.00] |
| **Poor sleep quality** | 0.95 [0.83-1.09] |
| **No mental health disorders** | 0.91 [0.85-0.98] |
| **No anxiety** | 0.93 [0.86-1.00] |
| **Anxiety disorders (current)** | 1.37 [0.86-2.17] |
| **No depression** | 0.93 [0.87-1.00] |
| **Depression (current)** | 0.92 [0.58-1.47] |
| **No schizophrenia** | 0.95 [0.88-1.02] |
| **Schizophrenia (lifetime)** | 1.32 [0.25-6.92] |
| **No bipolar disorder** | 0.95 [0.94-0.95] |
| **Bipolar disorder** | 1.40 [1.38-1.42] |
| **No neuroticism** | 0.99 [0.89-1.10] |
| **Neuroticism** | 0.85 [0.76-0.95] |


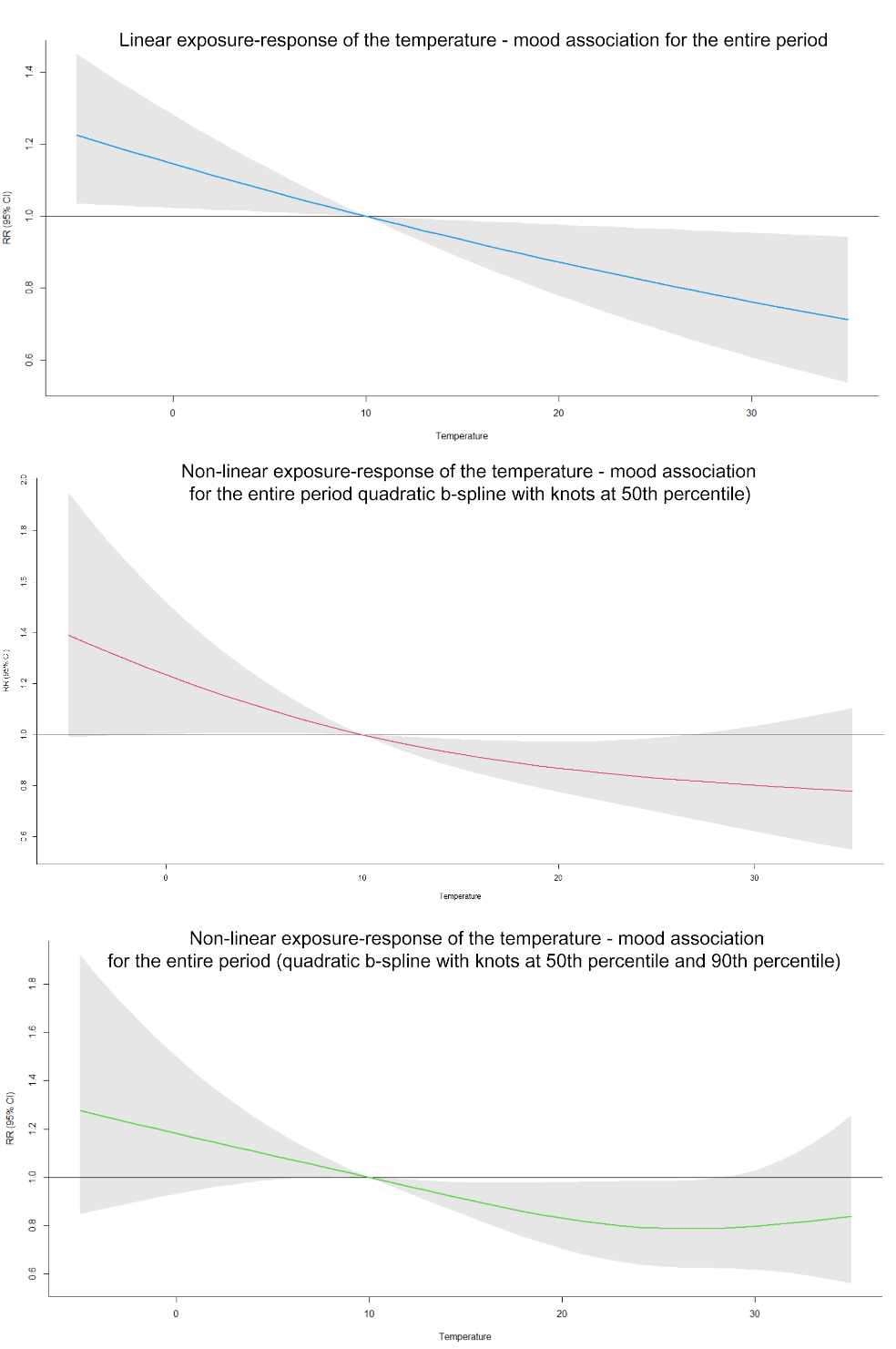


**Figure S1.** Sensitivity analysis: Comparison of the linear vs. non-linear exposure-response (quadratic b-spline with knots at 50^th^ and at the 50^th^ and 90^th^ percentile) of the temperature – mood associations for the entire study period.

**Table S5.** Sensitivity analyses: Association between bad mood and maximum temperature for wider and narrower radiuses of the weather station.

|  | **Odd ratio [95% confidence interval]** |
| --- | --- |
| Narrower radius (4 km) | 0.93 [0.88-0.99] |
| Wider radius (20 km) | 0.94 [0.89-0.99] |

**
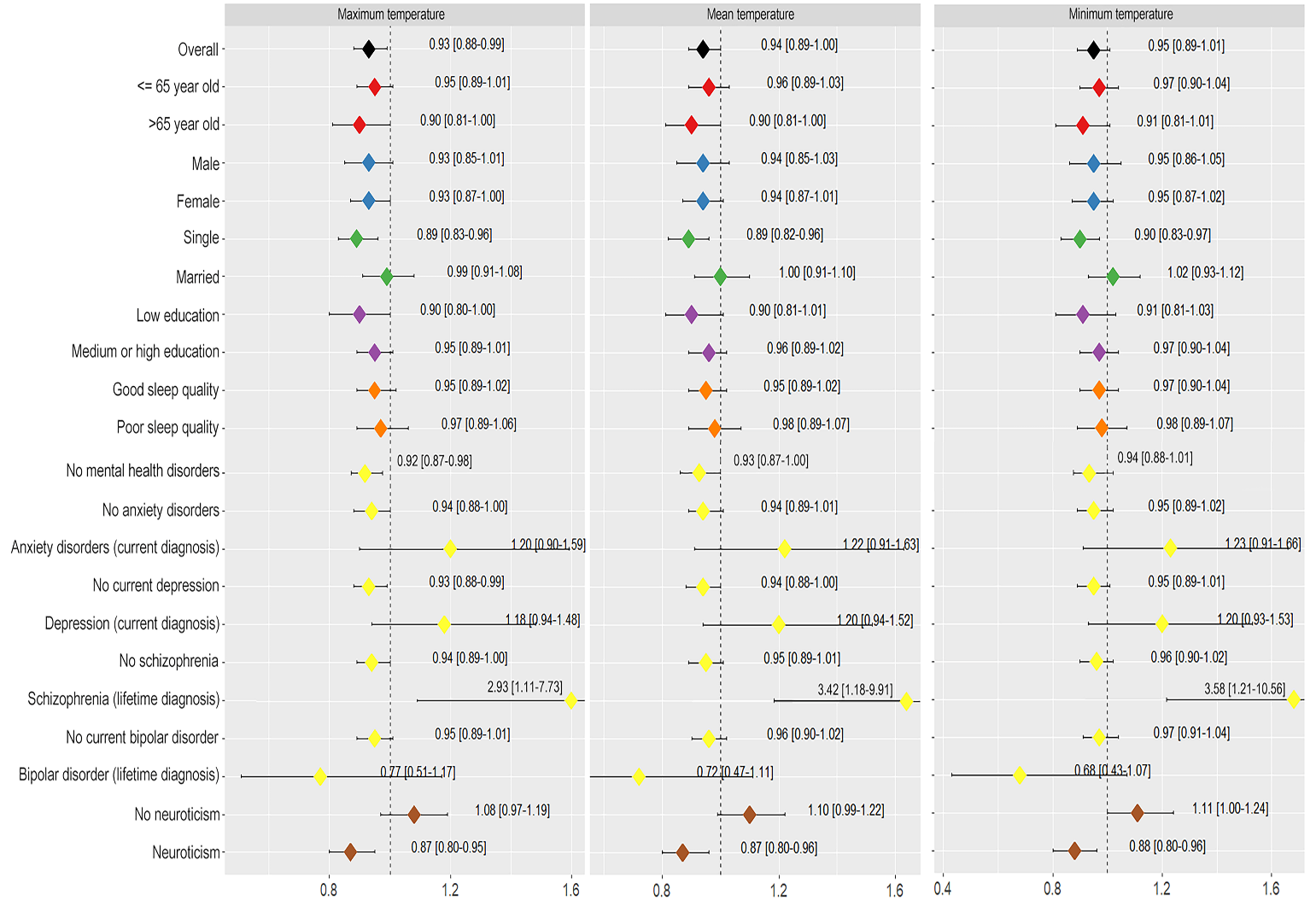
**

**Figure S2**. Sensitivity analyses: (odds ratio and 95% confidence interval) of average temperature, minimum temperature, maximum temperature and bad mood.

A result below 1 means that an increase in 5ºC of temperature reduces the probability of having a bad mood.

Anxiety disorder: agoraphobia, social phobia, panic disorder, general anxiety disorder

High neuroticism: score equal to or above the 50th percentile

## **References**

1. Preisig M, Waeber G, Vollenweider P, Bovet P, Rothen S, Vandeleur C, et al. The PsyCoLaus study: methodology and characteristics of the sample of a population-based survey on psychiatric disorders and their association with genetic and cardiovascular risk factors. BMC psychiatry. 2009;9:9.

2. Leboyer M, Barbe B, Gorwood P, Teherani M, Allilaire J, Preisig M, et al. Interview diagnostique pour les études génétiques. Inserm, Paris. 1995.

3. Nurnberger JI, Jr., Blehar MC, Kaufmann CA, York-Cooler C, Simpson SG, Harkavy-Friedman J, et al. Diagnostic interview for genetic studies. Rationale, unique features, and training. NIMH Genetics Initiative. Archives of general psychiatry. 1994;51(11):849-59; discussion 63-4.

4. Preisig M, Fenton BT, Matthey ML, Berney A, Ferrero F. Diagnostic interview for genetic studies (DIGS): inter-rater and test-retest reliability of the French version. European archives of psychiatry and clinical neuroscience. 1999;249(4):174-9.

5. Leboyer M, Maier W, Teherani M, Lichtermann D, D'Amato T, Franke P, et al. The reliability of the SADS-LA in a family study setting. European archives of psychiatry and clinical neuroscience. 1991;241(3):165-9.

6. American Psychiatric Association A, Association AP. Diagnostic and statistical manual of mental disorders: DSM-IV: American psychiatric association Washington, DC; 1994.
